# Removal of a frameshift between the *hsdM* and *hsdS* genes of the EcoKI Type IA DNA restriction and modification system produces a new type of system and links the different families of Type I systems

**DOI:** 10.1093/nar/gks876

**Published:** 2012-09-23

**Authors:** Gareth A. Roberts, Kai Chen, Laurie P. Cooper, John H. White, Garry W. Blakely, David T. F. Dryden

**Affiliations:** ^1^EastChem School of Chemistry, The University of Edinburgh, The King’s Buildings, Edinburgh, EH9 3JJ and ^2^Institute of Cell Biology, School of Biological Sciences, The University of Edinburgh, The King’s Buildings, Edinburgh EH9 3JR, UK

## Abstract

The EcoKI DNA methyltransferase is a trimeric protein comprised of two modification subunits (M) and one sequence specificity subunit (S). This enzyme forms the core of the EcoKI restriction/modification (RM) enzyme. The 3′ end of the gene encoding the M subunit overlaps by 1 nt the start of the gene for the S subunit. Translation from the two different open reading frames is translationally coupled. Mutagenesis to remove the frameshift and fuse the two subunits together produces a functional RM enzyme *in vivo* with the same properties as the natural EcoKI system. The fusion protein can be purified and forms an active restriction enzyme upon addition of restriction subunits and of additional M subunit. The Type I RM systems are grouped into families, IA to IE, defined by complementation, hybridization and sequence similarity. The fusion protein forms an evolutionary intermediate form lying between the Type IA family of RM enzymes and the Type IB family of RM enzymes which have the frameshift located at a different part of the gene sequence.

## INTRODUCTION

The phenomenon of restriction and modification (RM) is used by bacteria to control the influx of foreign DNA by horizontal gene transfer (1). RM systems are enzymes which maintain the sequence-specific methylation of defined target sequences on the host chromosome (modification) and recognize and cleave foreign DNA containing unmethylated target sequences (2–4). Thousands of RM systems have been discovered and grouped in to various classes named Types I, II and III (5,6). The simplest Type II RM systems comprise separate restriction endonucleases and DNA methyltransferases (MTase) but many Type II and all Type I and III RM systems combine the RM functions in one enzyme complex. These systems often have multiple subunits, each expressing individual endonuclease, MTase or sequence recognition functions, but sometimes are found as a single polypeptide (SP) in which the subunits and their individual activities are fused together. Examples would be the SP enzymes RM.BpuSI (a Type IIG) (7) and LlaGI (a Type ISP with SP meaning single polypeptide) (8) or the Type IIB enzyme BcgI which fuses endonuclease and MTase function but keeps sequence recognition as a separate subunit (9,10). Fusions have been created artificially for a number of Type II RM systems and functionality has been maintained (11,12).

**Figure 1.**
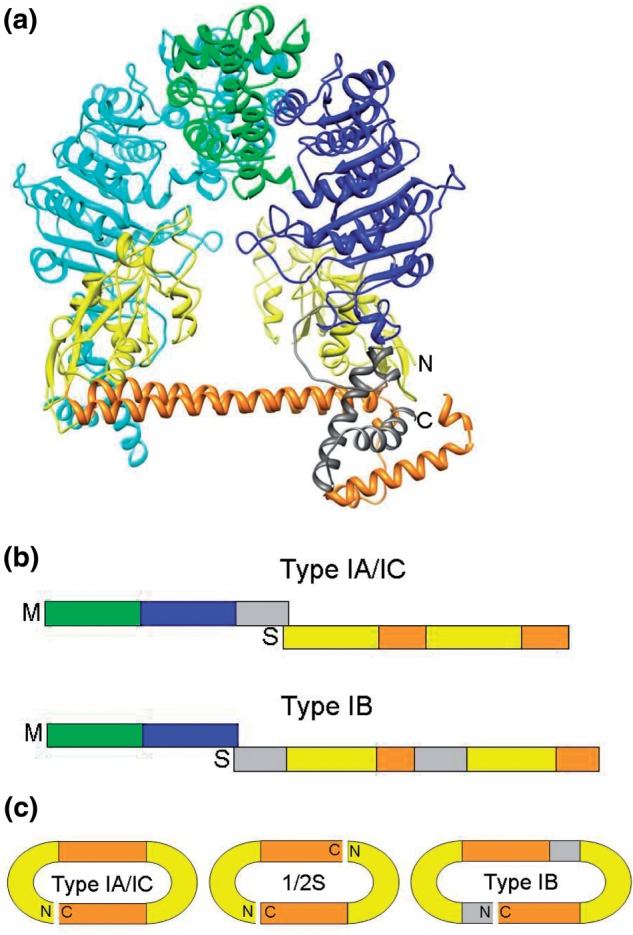
Structures of Type I MTases showing the HsdS subunit with TRDS (yellow) and conserved helical regions (orange), and the two HsdM subunits (one in cyan, the other with the N-terminal domain in green, the MTase catalytic domain in blue and the C-terminal helical tail in grey). (**a**) The EcoKI MTase structural model (14). The C terminus of an M subunit and the N terminus of the S subunit are indicated by ‘C’ and ‘N’, respectively, and are close together in space. (**b**) A schematic of the gene layout for typical Type IA/IC RM systems and for Type IB RM systems. The frameshift at the junction between regions coding for HsdM and HsdS subunits is indicated by showing the *hsdM* gene above the *hsdS* gene. The colour scheme is as in part (a) (with the additional inserts in the *hsdS* gene for the IB system coloured grey for reasons discussed later). A small conserved section at the start of *hsdS* is not shown for clarity. (**c**) The circular arrangement of HsdS subunit sequences for a Type IA/IC HsdS subunit, as originally proposed by Kneale (23), for a half-S subunit and for the Type IB HsdS subunit.

The Type I RM enzymes are active as a large multifunctional molecular machine containing two molecules of the HsdR (R) restriction endonuclease subunit, two molecules of the HsdM (M) modification MTase subunit and one molecule of the HsdS (S) sequence recognition subunit (13). An active MTase is also formed by two HsdM and one HsdS (14; Figure 1a and b). Doubly methylated targets on the host are resistant to the restriction endonuclease, hemimethylated DNA targets produced after chromosome replication are converted to fully methylated targets by the RM enzyme or the MTase and unmethylated target sequences are cleaved by the RM enzyme at a DNA site remote from the target sequence. The remote cleavage site is reached by ATP hydrolysis-dependent DNA translocation by motor domains in the HsdR. Cleavage occurs when translocation is blocked, usually by collision with a second translocating Type I RM enzyme *in vitro* (15–17), with an arrested DNA replication fork (18) and a Holliday junction *in vitro* (17) and, presumably, *in vivo*.

The HsdS subunit contains two target recognition domains (TRDs) each of which is responsible for recognizing one half of the bipartite DNA target (19–21), e.g. the EcoKI Type I RM system recognizes the sequence AACN_6_GTGC with methylation at the adenines at the underlined locations (22). The TRDs are separated by two alpha helices lying side by side in an anti-parallel arrangement and since the N and C termini lie nearby in one of these helices, then circular permutations of the gene and subunit are possible (23,24; Figure 1c). The number of repeating of amino acid sequences (25) in these alpha helices defines the number of nucleotides between the two half sites in the recognition sequence (26–29).

A further important feature of Type I RM systems is the existence of families defined by the ability to show antibody cross-reactivity, DNA hybridization and subunit complementation within a family but not between families (30,31). The Type IA, IC, ID and IE families possess HsdR, HsdM and HsdS subunits of roughly equivalent lengths but the Type IB family have smaller HsdM subunits and longer HsdS subunits than the other families (25). The Type IB HsdS subunits have extra long conserved regions. It was suggested that the IA and IB families (and by implication the other families), could be formed from an ancestral system via gene duplication of a primordial ‘half S’ (25). Movement of TRDs by recombination has also been proposed from an analysis of *Helicobacter pylori* genome sequences (32). It is possible to swap entire TRDs between Type I RM systems (33–35) as long as they are in the same family (30,31) or to create ‘half-S’ subunits with only one TRD and one alpha helix. These dimerise to produce functional Type I RM systems with symmetrical target recognition sequences (36–38).

An interesting feature of the genetic organization of the classical *Escherichia coli* Type I RM systems is that the *hsdR* usually has its own promoter whereas the *hsdM* and *hsdS* are often transcribed from a single promoter upstream of the *hsdM* (39). The junction between the *hsdM* and *hsdS* genes overlaps by a few base pairs and the genes are translated from different reading frames (Figure 1b). It has been proposed that translational coupling similar to that observed in the *trp* operon (40) occurs from one reading frame to another during translation of the *hsdM-hsdS* mRNA and this leads to the two separate polypeptides being formed. The structural model (13,14), Figure 1a suggests that the alpha helical C terminal region of the HsdM subunit lies alongside the alpha helical region between the TRDs of the HsdS subunit in such a manner so that the end of each HsdM polypeptide is near the start of each TRD of the HsdS.

In this article we investigate the effect of removing the frameshift between the *hsdM* and *hsdS* genes of the EcoKI Type I RM on RM activity *in vivo* and *in vitro*. We show that the fusion protein supports both restriction and modification *in vivo* in the presence of HsdR. It recognizes the same target sequence as EcoKI and no other target. *In vitro* the fusion protein is stable and can support DNA cleavage activity in the presence of HsdR but only after additional HsdM subunit was added. This suggests that either some proteolysis of the fusion occurs or the translation process still occasionally stops at the end of *hsdM* to provide a pool of HsdM subunit *in vivo*. Irrespective of whether proteolysis or translation is important, the fact that a gene fusion in a Type I RM system produces an active and new type of RM system has implications for the appearance of diverse RM systems. By amino acid sequence comparisons we show that the fusion can represent an intermediate form leading to the formation of the different families of Type I RM systems.

## MATERIALS AND METHODS

### Chemicals, bacterial strains and phage

All chemicals were purchased from Sigma-Aldrich unless otherwise stated.

Bacterial strains *E. coli* NM679 (r^−^m^−^s^−^), *E. coli* NM1049 (r^+^m^+^s^+^) and *E. coli* NM1261 (r^+^m^+^s^−^) were used (41,42). DE3 lysogens of NM1049 and NM1261 were prepared as described previously (43) and used in all *in vivo* assays.

The pJFMS plasmid for expression of EcoKI MTase has been previously described (44). The pMSFusion plasmid is derived from pJFMS. The *hsdM* open reading frame was fused in frame to *hsdS* by the polymerase chain reaction (PCR) and the resulting product was ligated into the parental vector. PCR using oligonucleotides ‘pJFMS promoter’ (5′ GCTTCTGGCGTCAGGCAGCC 3′) and ‘MSfusionBS’ (5′ CCGGCAATTTCCCCGCACTCATTTCCTTCACCCCACCAAACGC 3′), with pJFMS as template, generated a fragment comprising the 5′ UTR upstream of *hsdM* and the entire *hsd*M ORF, fused in frame with the first 22 bases of *hsdS* by the insertion of a single adenine base. Similarly, PCR with the second pair of oligonucleotides, ‘MSfusionTS’ (5′ GCGTTTGGTGGGGTGAAGGAAATGAGTGCGGGGAAATTGCCGG 3′) and ‘SwildtypeCterm’ (5′ GGATCCGAATTCTCAGGATTTTTTACGTGAGGC 3′), with pJFMS as template, generated a fragment comprising the last 20 bases of *hsdM* fused to the entire ORF of *hsdS* by the insertion of a single adenine base. These two PCR products were purified and fused in a reaction primed with oligonucleotides ‘pJFMS promoter’ and ‘SwildtypeCterm’. The resulting product was purified and digested with SmaI and EcoRI. pJFMS was digested with SmaI and EcoRI, treated with Calf Intestinal Phosphatase and then ligated with the PCR product. Recombinant plasmids were isolated from transformed *E. coli* DH5α cells and the desired DNA sequence was confirmed. Orientation of the insert and continuity of the *hsdS* reading frame were confirmed by DNA sequencing. This plasmid was named pMSFusion and we call the protein MSFusion.

The pBIO2 plasmid is a negative control construct derived from pJFMS and contains most of the *hsdS* ORF with a C-terminal fused biotinylation signal. It has no start codon and therefore does not encode a functional MTase. The insert is derived from pJFMS by PCR (oligos: ‘OligohsdM1’ 5′ CATCACTTTCTGCTGGTGC 3′ and ‘hsdMBiotincterm’ 5′ GAACTTGAATTCTTATTCGTGCCATTCGATTTTCTGAGCCTCGAAGATGTCGTTCAGACCGCCACCGGATTTTTTACGTGAGGCTTTTTTACCCCC 3′) and was ligated into the HindIII-EcoRI interval of the vector fragment of pJFMS.

The pBS17 plasmid contains the EcoKI *hsdR* gene ligated into the DraI interval of pACYC184 as a BfrBI-SmaI fragment. The R subunit was expressed from its natural promoter, function being confirmed by restoration of restriction in strain *E. coli* DH5α (data not shown).

### Assessment of RM activity *in vivo*

The methods employed for assaying RM activity *in vivo* used the efficiency of plating (eop) of phage lambda (virulent) λv.k or λv.o (k for EcoKI modified, o for unmodified) on the various strains of *E. coli* have been previously described (43,45). All assays were performed as spot tests in triplicate and the eop error is ∼25%. The promoter on the expression plasmids is slightly leaky so IPTG was not required to be added to the plates.

### Expression and purification of proteins

EcoKI MTase (M_2_S_1_), the partially assembled form M_1_S_1_, HsdM, HsdR and EcoKI nuclease were prepared as described previously (16,44).

Overexpression of the MSFusion protein from pMSFusion was performed after transforming *E. coli* BL21(DE3) and induction of expression by adding IPTG to 1 mM and further growth at 37°C for 4 h. To prepare the MSFusion protein, ∼15 g of cell pellet was defrosted on ice and resuspended in 50 ml of buffer A (20 mM Tris–HCl, 20 mM 2-(N-morpholino)ethanesulfonic acid (MES), 10 mM MgCl_2_, and 7 mM 2-mercaptoethanol, pH 8). A protease inhibitor tablet was added to the buffer (complete protease inhibitor—Roche). The cells were disrupted by sonication on ice using a Soniprep 150 sonicator (Sanyo, Tokyo, Japan) fitted with a 9 mm diameter probe for ∼15 min with intermittent cooling between bursts.

The sonicated cells were centrifuged at 20 000 g for 1 h at 4°C, and the supernatant filtered through a filter unit (0.45 um; Sartorius AG, Goettingen, Germany). The clarified extract was then applied to a DEAE anion exchange column (30 cm × 1.6 cm diameter), equilibrated with buffer A, at a flow rate of 60 ml/h. The column was washed with approximately three column volumes of buffer A to remove unbound material. Bound proteins were then eluted using a 500 ml linear gradient of 0–0.8 M NaCl in buffer A at a flow rate of 20 ml/h. Fractions giving a UV absorbance were then analysed by SDS-PAGE. The fractions containing the MSFusion protein were pooled and dialyzed against 2 L of buffer A for 3 h to remove the vast majority of the NaCl. At this stage some of the protein precipitated and was removed. (This precipitation event was found to be partially reversible on addition of salt solution.)

The remaining soluble protein after dialysis was applied to a heparin agarose affinity column (15 cm × 1.6 cm diameter), equilibrated with buffer A, at a flow rate of 50 ml/h. This was then washed with approximately two column volumes of buffer A to remove unbound material. Bound proteins were eluted using a 500 ml linear gradient of 0–0.8 M NaCl in buffer A at a flow rate of 20 ml/h. Fractions giving a UV absorbance were analysed by SDS-PAGE. The protein was pooled into two lots depending on its purity. The purest sample was concentrated using a spin concentrator with a 30 kDa cutoff membrane (VivaScience AG, Hanover, Germany). The protein was stored at −20°C in buffer A containing 50% (v/v) glycerol. The final yield was ∼27 mg of >95% pure protein. Molecular weight markers for the SDS-PAGE were prestained Precision Plus Protein Dual Color standards (Biorad).

Assuming the MSFusion protein is a monomer, an extinction coefficient of 84289 M^−^^1^ cm^−^^1^ was obtained using the online Protein Calculator program (http://www.scripps.edu/∼cdputnam/protcalc.html). Coefficients calculated in this manner are accurate to ±5% (46).

### *In vitro* DNA cleavage assay

The *in vitro* endonuclease assay monitored the linearization of unmethylated plasmid pBRsk1 by EcoKI, EcoKI MTase plus HsdR, and the MSFusion with added HsdM and HsdR. The plasmid has a single target site for EcoKI (47). Typically, the reaction was performed in a 50 µl reaction volume containing 10 mM Mg-acetate, 10 mM Tris-acetate, 7 mM 2-mercaptoethanol, 50 µg/ml BSA (New England Biolabs), 2 mM ATP, 0.1 mM S-adenosyl methionine (SAM, New England Biolabs), 3 nM pBRsk1 and initiated by adding protein to 30 nM. Where required, proteins and subunits were mixed for 2 min at room temperature prior to addition of the other reaction components. Digests were performed for 8 min at 37°C before stopping by incubation at 68°C for 10 min. The extent of reaction was then analysed by agarose gel electrophoresis on a 0.8% TAE gel. DNA bands were visualized under UV illumination after staining with ethidium bromide.

### Western blots

Antibodies used have been described previously (48). All protein samples were run on SDS-PAGE gels and blotted onto PVDF membrane (Bio-Rad) for 2 h at 60 V in 20 mM Tris–HCl pH 8, 150 mM glycine, 20% (v/v) methanol buffer. The membrane was blocked with a 5% milk solution and then incubated with the primary antibodies overnight. The membrane was washed several times in phosphate buffered saline and then incubated with the secondary antibody anti-rabbit AP conjugate (Promega) for 1 h. After several more washing cycles with phosphate buffered saline, the reacted bands were visualized using the alkaline phosphatase buffer/colour development method from Promega (nitro-blue tetrazolium and 5-bromo-4-chloro-3′-indolyphosphate). Molecular weight markers for the blot were prestained Precision Plus Protein Dual Color standards (Biorad).

### Sequence analysis

Amino acid sequences for the HsdS and HsdM subunits of EcoKI and EcoR124I were obtained from REBASE (6). BLASTP (49) was used for sequence comparisons and Chimera (50) for structure visualization.

## RESULTS

### Complementation of an r^+^m^+^s^−^ EcoKI RM strain, *E. coli* NM1261(DE3), with pMSFusion

**Figure 2.**
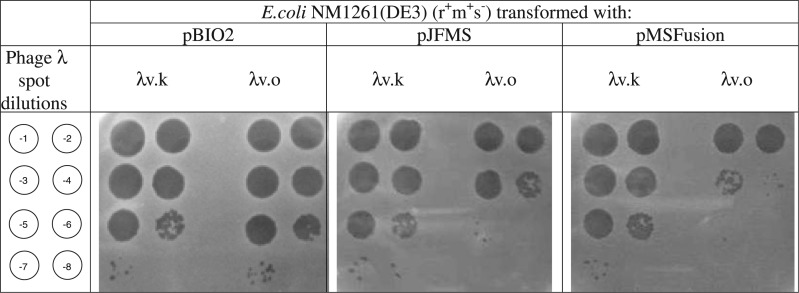
*Escherichia coli* NM1261(DE3) (r^+^m^+^s^−^) transformed with, from left to right, plasmids pBio2, pJFMS and pMSFusion. Phage were then spotted on these strains with λv.k on left and λv.o on right of each panel and dilutions from 10^−1^ to 10^−8^ as indicated by the diagram at the left.

*Escherichia coli* NM1261(DE3) lacks the gene for the HsdS subunit but does express both HsdR and HsdM subunits, a genotype of r^+^m^+^s^−^. These cells were transformed with either the pBIO2 vector, pJFMS expressing the EcoKI MTase or the pMSFusion expressing the fusion protein. As expected, λv.k plated well on all three strains as it is resistant to restriction by EcoKI (Figure 2; an eop of ∼1). λv.o plated well on cells transformed with pBIO2 as expected but was severely restricted when plated on cells containing pJFMS (an eop of 2.7 × 10^−^^3^) indicating that its EcoKI sites were unmethylated and that the HsdS subunit expressed from the plasmid was able to complement the HsdR and HsdM subunits expressed from the chromosome or the plasmid (Figure 2). λv.o also plated very poorly on cells transformed with pMSFusion (an eop of 5.4 × 10^−^^4^; Figure 2). Restriction in cells transformed with pMSFusion was consistently greater than restriction in cells transformed with pJFMS. This can most probably be attributed to a variation in protein expression level. This result demonstrates that the protein is being expressed by pMSFusion and is able to complement the HsdR and HsdM subunits being expressed from the chromosome.

### Restriction in an r^−^m^−^s^−^ EcoKI RM strain, *E. coli* NM679, with pMSFusion and pBS17 expressing HsdR

*Escherichia coli* NM679 has the genes for EcoKI completely deleted. Transformation of this strain with pJFMS and pBS17 leads to expression of the EcoKI system and ∼10^4^-fold reduction in the eop of λv.o when compared to the strain transformed with pJFMS alone (an eop of ∼1x10^−^^4^; Supplementary Figure S1). Transformation of this strain with pMSFusion and pBS17 leads to expression of the fusion protein and the HsdR subunit and ∼10^3^-fold reduction in the eop of λv.o when compared to the strain transformed with pMSFusion alone (an eop of ∼1 × 10^−^^3^; Supplementary Figure S1). This indicates that the fused gene and the *hsdR* gene are sufficient for restriction of λv.o; no extra *hsdM* gene is required.

### The absence of hsdR results in no restriction

The eop of phage λv.k and λv.o on *E. coli* NM679 transformed with pJFMS or pMSFusion was measured (Supplementary Figure S2). No difference in plating efficiency was observed indicating that pMSFusion is not expressing any unexpected nuclease activity.

### Phages are modified by cells transformed with pMSFusion

**Figure 3.**
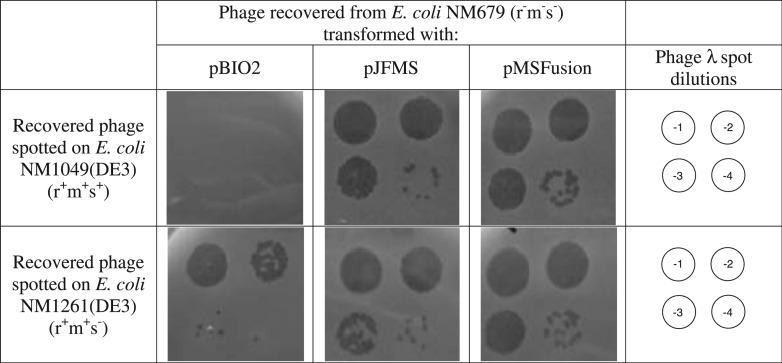
Phage recovered from *E. coli* NM679 spotted on *E. coli* NM1049(DE3) (r^+^m^+^s^+^), top row, or on *E. coli* NM1261(DE3) (r^+^m^+^s^−^), bottom row). From left to right are cells transformed with pBIO2, pJFMS or pMSFusion. Phage dilutions are 10^−1^ to 10^−4^ as shown.

Phage were recovered from *E. coli* NM679 transformed with pBIO2, pJFMS or pMSFusion. *E. coli* NM1049(DE3) expresses EcoKI from the chromosome (r^+^m^+^s^+^). As expected the phage recovered from cells transformed with pBIO2 vector where severely restricted on *E. coli* NM1049(DE3) but not on *E. coli* NM1261(DE3) (Figure 3). Phage recovered from cells transformed with pJFMS were resistant to restriction on *E. coli* NM1049(DE3) as expected because they were methylated by passage through the cells transformed with pJFMS (Figure 3). Phage recovered from cells transformed with pMSFusion were resistant to restriction on *E. coli* NM1049(DE3) (Figure 3). This indicates that they had become modified by passage through cells transformed with pMSFusion and thus the fusion protein is capable of EcoKI-specific modification.

### The fusion protein methylates only EcoKI target sites

Phage previously modified by EcoKI, λv.k, were plated on *E. coli* NM679 transformed with pBS17 and either pJFMS or pMSFusion. Phage plated on cells transformed with pBS17 showed no restriction as expected (Supplementary Figure S3). Similarly phage plated on cells transformed with pBS17 and pJFMS, and thus expressing active EcoKI, were not restricted as their target sites were already modified (Supplementary Figure S3). Phage is also not restricted when plated on cells transformed with pBS17 and pMSFusion (Supplementary Figure S3). This indicates that λv.k does not contain any additional target sites for the fusion protein, in other words; the target specificity of the fusion protein and the normal HsdS subunit of EcoKI are the same.

### Expression and purification of the MSFusion protein and its interactions with HsdM, HsdR and DNA

Induction of expression of MSFusion protein was easily accomplished and the protein could be purified in large quantities and stored stably in glycerol at low temperatures for months. Supplementary Figure S4 shows SDS-PAGE gels for each step of the purification.

### *In vitro* nuclease activity of the MSFusion protein requires additional HsdM subunits

**Figure 4.**
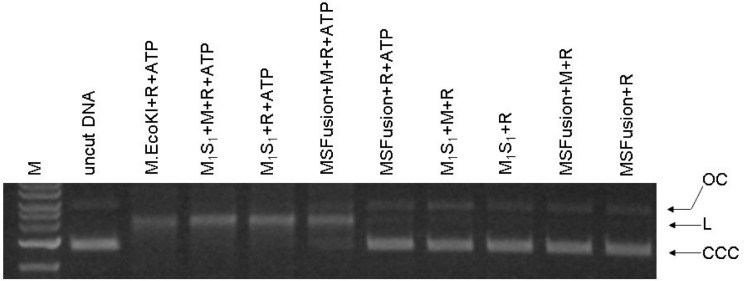
Endonuclease assay for DNA cleavage by MSFusion. Final concentration of DNA (unmethylated pBRsk1) was 3 nM. Final concentration of reconstituted nuclease was 60 nM using at least 2 HsdR and 1 HsdM per M_1_S_1_ protein or MSFusion protein. The enzymes were reconstituted at 1000 nM and then diluted into the solution containing DNA. M = 1 kb ladder; OC = open circle; L = linear; CCC = covalent closed circle.

Sufficient quantities of HsdR were available for nuclease DNA cleavage assays but not for more detailed structural analysis. Mixtures of HsdR with EcoKI MTase, M_1_S_1_ ±HsdM and MSFusion ±HsdM were examined for their ability to cut a plasmid containing a single unmodified EcoKI target site (Figure 4). As expected, ATP was found to be essential for observation of any cleavage of the circular plasmid to a linear form. Mixing M_2_S_1_ with HsdR or M_1_S_1_ with HsdR showed cleavage activity. Recalling that the M_1_S_1_ preparation includes some M_2_S_1_, this cleavage activity is expected. Adding additional HsdM to the M_1_S_1_ + HsdR mixtures did not cause extra cleavage. However, mixing HsdR with MSFusion did not give observable DNA cleavage unless HsdM was also added. This contradicts the *in vivo* observation, Supplementary Figure S1, that HsdR and MSFusion together are sufficient to give restriction and modification (i.e. *hsdM* is not required *in vivo*).

### Source of free HsdM *in vivo*

The above results indicate a discrepancy between *in vivo* restriction where *hsdM* is not required and *in vitro* nuclease activity where HsdM is required. Free HsdM may be made *in vivo* in the absence of *hsdM* by proteolysis of the MSFusion protein or by translational stalling while synthesising MSFusion.

**Figure 5.**
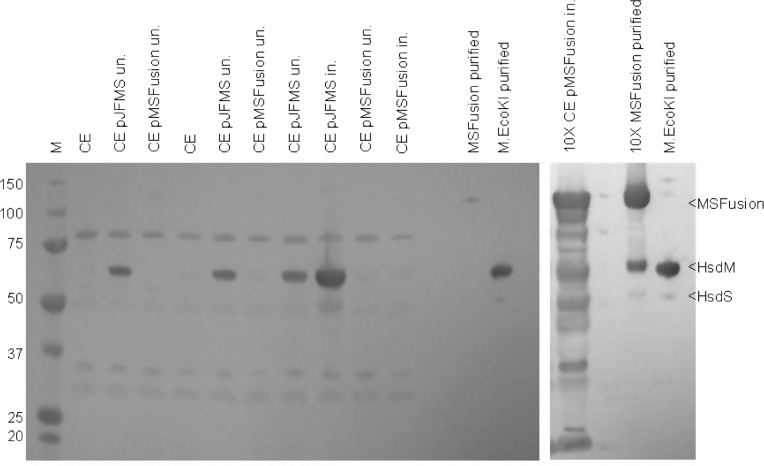
Western blot of cell extracts using antibodies specific to HsdM and HsdS to detect the MSFusion protein and the presence of any HsdM produced by proteolysis or stalled translation of the fusion protein. M = molecular weight marker; CE = cell extract of *E. coli* NM679; un. = uninduced; in. = induced.

The presence of free HsdM in cell extracts was examined using western blotting and antibodies against the EcoKI MTase HsdM and HsdS subunits. The blotting experiment showed the presence of HsdM and HsdS in EcoKI control cell extracts (Figure 5 lanes 2, 5, 7 and 8). However, no HsdM was visible in a cell extract from cells expressing large amounts of the MSFusion protein (Figure 5 lanes 9, 10 and 13 nor indeed was any MSFusion). The western blot indicated that the antibody signal from the fusion protein itself was weak, lanes 9–11, even though a large amount of protein was visible when stained with Coomassie Blue suggesting that the best epitopes for the polyclonal antibodies are obscured in the MSFusion protein. When an excess of purified MSFusion was used, lane 14, not only was the fusion protein observed but a weak signal at the molecular weight expected for HsdM was also visible. If HsdM or fragments of HsdM where being produced by proteolysis or translational stalling then some signal would have been expected though unfortunately the antibodies are rather non-specific and generate a signal for many proteins of ∼60 kD in the extract, Figure 5 lane 13, thus a signal from HsdM may be obscured in these cell extracts. Given that the *in vitro* nuclease assays indicate that HsdM must be present for activity, the apparent absence of HsdM in cell extracts must indicate that they are present but at extremely low levels. This suggests that a small proportion of HsdM is produced from pMSFusion and copurifies with the fusion protein. While there is not enough of this co-purifying HsdM in the ‘pure’ MSFusion sample to generate a nuclease activity visible in Figure 4, there is probably sufficient to generate nuclease activity *in vivo*. Thus the apparent contradiction between the *in vivo* restriction results and the *in vitro* nuclease results can be reconciled.

## DISCUSSION

### The fusion protein as a new RM system: the Type IMS RM system

The new RM system created in this work requires a name and we suggest Type IMS with ‘MS’ for fusion of HsdM and HsdS and we would apply this name to proteins containing either two TRDs or a single TRD and a separate HsdR subunit.

The fusion of two subunits, HsdM and HsdS, of EcoKI produced a viable RM system with the same sequence specificity as the original EcoKI RM system. The new RM system was still able to restrict and modify DNA. Although the presence of a separate HsdM subunit was not apparently required for RM activity of the new enzyme *in vivo*, *in vitro* experiments showed that HsdM was required. Thus the new RM enzyme would most probably be formed by two HsdR, one HsdM and one MSFusion protein to match the R_2_M_2_S_1_ stoichiometry of normal EcoKI. The missing HsdM subunit *in vivo* could be produced *in vivo* by proteolysis of the fusion protein or by translational stalling.

It is also possible that the RM system *in vivo* may be formed from two HsdR and two MSFusion in the absence of any extra HsdM subunits although this seems less likely given the results from the *in vitro* nuclease assay. Such an assembly would have an ‘extra’ copy of the HsdS subunit and this presumably would have to be pushed out of the way when assembling the RM enzyme. The presence of multiple TRDs, some of which must be pushed out of the way when assembling an active enzyme, is not without precedent as some *H. pylori* Type I RM systems appear to have three TRDs in their HsdS (32) and some Type II MTases contain as many as five, consecutive, functional TRDs (though only one TRD is active at a time) (51). However given the results of the western blot (Figure 5), we believe that the presence of some HsdM is the most likely reason for activity.

These results show that a simple frameshift in open reading frames in the EcoKI Type I RM system and can set it on the path to evolve new families of Type I RM systems. The success of such a trivial change is remarkable from a structural point of view and indicates that the C-terminus of HsdM must be in close proximity to the N-terminal end of the HsdS supporting the published model of the structure of the EcoKI MTase and RM enzyme (13,14).

### Evolution of Type IB RM enzymes from Type IA/IC RM enzymes

**Figure 6.**
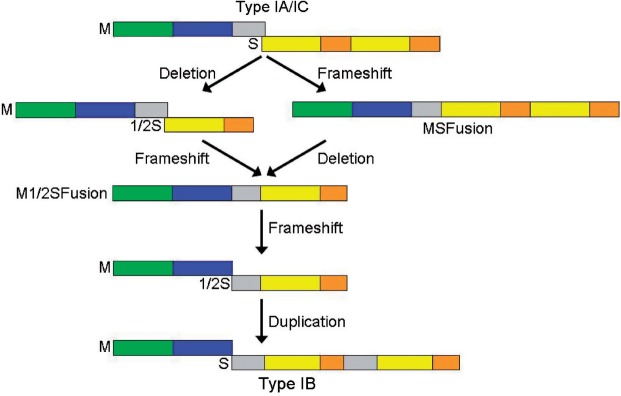
Two possible evolutionary routes to convert between Type IA/IC RM systems and Type IB RM systems via a Type IMS RM system with HsdM subunit fused to HsdS subunit. The colouring scheme for structural domains is the same as in Figure 1.

The essential difference in the organization of the *hsdM* and *hsdS* genes between Type IB RM systems and the others is the location of the frameshift; it occurs earlier in the DNA sequence of the *hsdM-hsdS* region in a Type IB RM system. If we suggest that the DNA encoding the C-terminal tail of the HsdM subunit of a Type IA or IC system has been incorporated by a frameshift into the DNA encoding the HsdS subunit and subsequently duplicated then two simple evolutionary paths from the IA or IC families to the IB family via a Type IMS system can be postulated (Figure 6). With the results presented in this work, all of the evolutionary forms in this scheme have been found in nature or created by genetic engineering.

If the process postulated in Figure 6 is correct then the amino acid sequence of the tails of HsdM subunits from IA and IC families should be similar to the extra inserted sequences in the N-terminal and central conserved regions of HsdS subunits of Type IB systems (the grey boxes in the HsdS subunit of the Type IB system shown in Figure 6). One way to examine this possibility is to take a fused ‘artificial’ sequence of the C-terminus of HsdM with the N-terminus of HsdS of the EcoKI Type IA system or the EcoR124I Type IC system and look for such sequences in current databases. Although as expected the sequence searches produced predominantly alignments to either HsdM subunit C-termini or to HsdS subunit N-termini, several putative HsdS subunit sequences from the NCBI database showed good alignment of their central conserved region to the entire length of the artificial sequences (Supplementary Figure S5). The EcoKI fused sequence aligns with the central conserved region of the putative HsdS subunit of *Fusobacterium nucleatum* subsp. polymorphum ATCC 10 953 with 23% identity and 43% similarity. The EcoR124I fused sequence aligns with the central conserved regions of the putative HsdS subunits of *Xylella fastidiosa* Ann-1 (49% identity and 57% similarity), *Shewanella putrefaciens* CN-32 (49% identity and 64% similarity), and *Lactobacillus delbrueckii* subsp. Lactis (43% identity and 63% similarity). We assert that the success of these sequence searches in finding sequences in central conserved regions of HsdS subunits with similarity to the region at the boundary between HsdM and HsdS subunits in Type IA and IC RM systems supports the proposal that Type IB RM systems arose by the path shown in Figure 6.

The Type ISP RM systems such as LlaGI carry the gene fusion idea further than presented in this article as they have fused the HsdR to the MSFusion albeit using an MSFusion with only a single TRD (8). It would be of interest to create such a fusion from a classical Type I RM system such as EcoKI. Furthermore, the fused forms of Type I RM enzymes are moving ever closer to the IIB and IIG subclasses of Type II RM systems (5,7–12).

In conclusion, our results show that a simple frameshift in open reading frames can be removed from the Type I RM systems and set them on the path to evolve new types of RM systems. The success of such a trivial change is remarkable from a structural point of view and indicates that the C-terminus of HsdM must be in close proximity to the N-terminal end of the HsdS supporting the published model of the structure of the EcoKI MTase and RM enzyme (13,14).

## SUPPLEMENTARY DATA

Supplementary Data are available at NAR Online: Supplementary Figures 1–5.

## FUNDING

Funding for open access charge: Wellcome Trust.

*Conflict of interest statement*. None declared.

## Supplementary Material

Supplementary Data
