# Supplementary material for: Removal of a frameshift between the hsdM and hsdS genes of the EcoKI Type IA DNA restriction and modification system produces a new type of system and links the different families of Type I systems
Source: Nucleic Acids Res. 2012 Sep 23;40(21):10916–24. doi: 10.1093/nar/gks876 (PMC3510504; doi:10.1093/nar/gks876)
Supplement: Supplementary Data [file supp_40_21_10916__index.html]

Removal of a frameshift between the hsdM and hsdS genes of the EcoKI Type IA DNA restriction and modification system produces a new type of system and links the different families of Type I systems — Removal of a frameshift between the hsdM and hsdS genes of the EcoKI Type IA DNA restriction and modification system produces a new type of system and links the different families of Type I systems — Supplementary Data 

# Removal of a frameshift between the *hsdM* and *hsdS* genes of the EcoKI Type IA DNA restriction and modification system produces a new type of system and links the different families of Type I systems

## Supplementary Data

files

**Files in this Data Supplement:**

- Supplementary Data - pdf file
