# Supplementary material for: Removal of a frameshift between the hsdM and hsdS genes of the EcoKI Type IA DNA restriction and modification system produces a new type of system and links the different families of Type I systems
Source: Nucleic Acids Res. 2012 Sep 23;40(21):10916–24. doi: 10.1093/nar/gks876 (PMC3510504; doi:10.1093/nar/gks876)
Supplement: Supplementary Data [file supp_gks876_nar-01881-v-2012-File003.pdf]

Supplementary data

Removal of a frameshift between the *hsdM* and *hsdS* genes of the EcoKI Type IA DNA restriction-modification (RM) system produces a new type of RM system and explains the origins of the different families of Type I RM systems.

Gareth A. Roberts[1, +], Kai Chen[1, +], Laurie P. Cooper[1, +], John H. White[1, +], Garry W. Blakely[2], David T.F. Dryden[1, *]

[1] EastChem School of Chemistry, The University of Edinburgh, The King's Buildings, Edinburgh, EH9 3JJ, UK.

[2] Institute of Cell Biology, School of Biological Sciences, The University of Edinburgh, The King's Buildings, Edinburgh EH9 3JR, UK.

Supplementary Figure S1. *E. coli* NM679 ($r^-m^-s^-$) transformed with pJFMS and pBS17 (R subunit) or pMSFusion and pBS17. Phage λv.o at various dilutions was then spotted on each strain.

	E. coli NM679 ($r^-m^-s^-$) transformed with:			
Phage λ spot dilutions	pJFMS	pJFMS + pBS17	pMSFusion	pMSFusion + pBS17
-1 -2<br>-3 -4<br>-5 -6<br>-7 -8	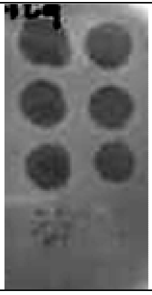	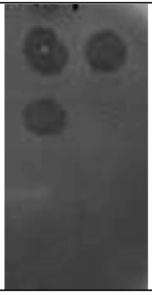	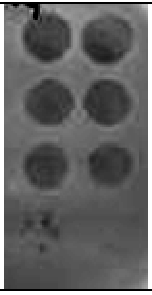	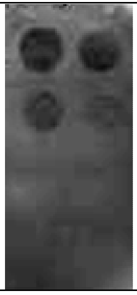

Supplementary Figure S2. *E. coli* NM679 ($r^-m^-s^-$) transformed with pJFMS or pMSFusion. Phage λv.k and λv.o were then spotted on these strains at various dilutions.

	E. coli NM679 ($r^-m^-s^-$) transformed with:			
	pJFMS		pMSFusion	
Phage λ spot dilutions	λv.k	λv.o	λv.k	λv.o
-1 -2<br>-3 -4<br>-5 -6<br>-7 -8	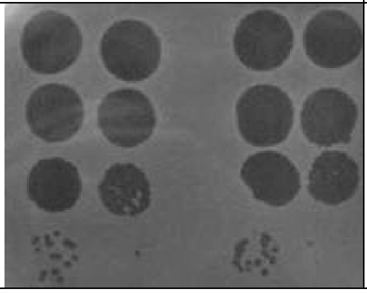		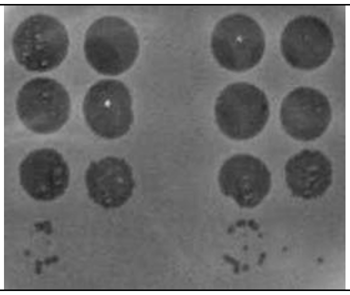	

Supplementary Figure S3. *E. coli* NM679 ($r^-m^-s^-$) transformed with plasmids pBS17 (HsdR subunit) and pJFMS, pBS17 and pMSFusion, and with pBS17 alone, was spotted with phage λv.k. The eop was identical for pBS17+pJFMS, pBS17+pMSFusion and pBS17.

E. coli NM679 ($r^-m^-s^-$) transformed with:			
pBS17 + pJFMS	pBS17 + pMSFusion	pBS17	Phage λ spot dilutions
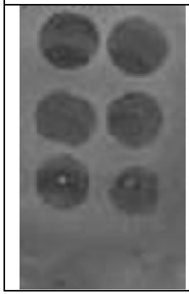	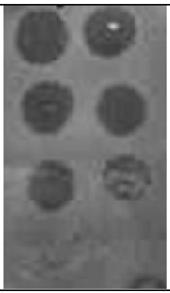	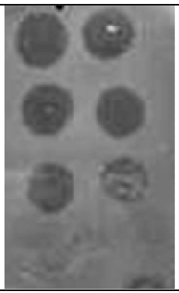	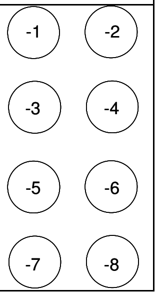

Supplementary Figure S4. Purification of MSFusion by column chromatography as visualised by Coomassie Blue staining of SDS-PAGE gels.

250,150,100,75,50,37,25,20,15,10

Purification step 1 - DEAE Column. M = Marker (250, 150, 100, 75, 50, 37, 25, 20, 15, 10 kDa), CE = Cell Extract, Lanes 1-12 are elution fractions from the salt gradient. Pooled fractions 3 and 4 for the heparin column.

M CE 1 2 3 4 5 6 7 8 9 10 11 12

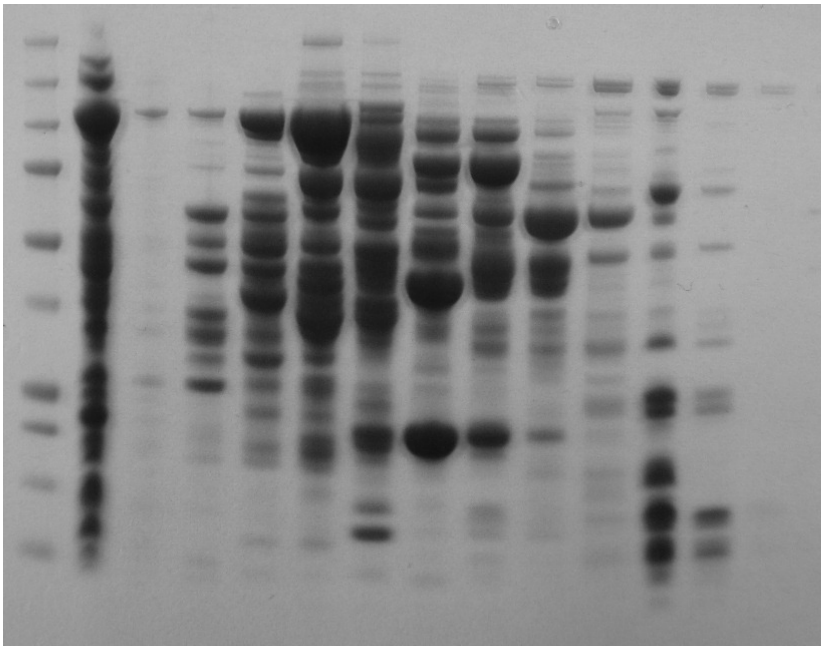

Purification step 2 - Heparin Affinity Column. M = Marker (kDa as above), D = DEAE pooled sample, FT = unbound material from heparin column. Lanes 1-12 are elution fractions from the salt gradient. Pooled fractions 8 to 11 as pure MSFusion.

M D FT 1 2 3 4 5 6 7 8 9 10 11 12

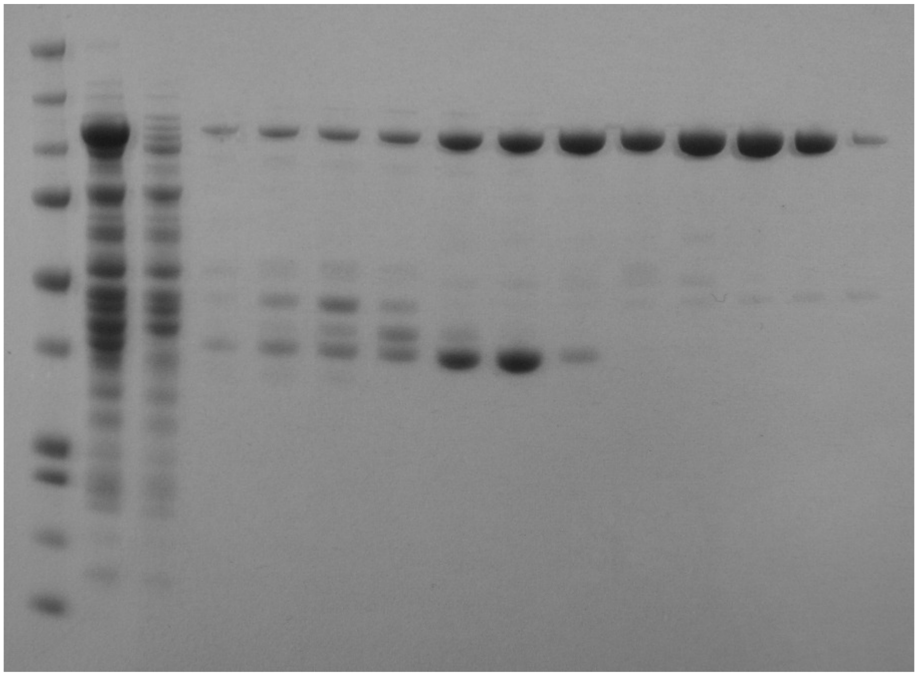

Supplementary figure S5. a. Amino acid sequences of the C-terminus of M subunit fused to N-terminus of S subunit for EcoKI and EcoR124I. The sequence in red is from the M subunit and in black from the S subunit. b. BLAST output using the fused EcoKI sequence from part a. pulls out the central conserved region of an S subunit in Fusobacterium nucleatum. c. BLAST output using the fused EcoR124I sequence from part a. pulls out the central conserved region of several S subunits from different bacteria.

a.

```
>MS.EcoKI
DSEENKNTDQ HLATSRWRKF SREWIRTAKS DSLDISWLKD KDSIDADSLP EPDVLAAEAM GELVQALSEL DALMRELGAS DEADLQRQLL
EEAFGGVKE MSAGKLPEGW VIAPVSTVTT LIRGVTYKKE QAINYLKDDY LPLIRANNIQ NGKFDTTDLV FVPKNLVKES QKISPEDIVI
AMSSGSKSVV GKSAHQHLPF

>MS.EcoR124I
QVFASKEDVA HLAKSVAFET VVANDYNLSV SSYVEAKDNR EIIDIAELNA ELKTTVSKID QLRKDIDAIV AEIEGCEVQK MSEMSYLEKL
LDGVEVEWLP LGEITKYEQP TKYLVKAKDY HDTYTIPVLT AGKTFILGYT NETHGIYQAS KAPVIIFDDF TTANKWVDFD FKAKSSAMKM
```

b.

```
ref|ZP_02273165.1|type I restriction modification enzyme protein S [Fusobacterium
nucleatum subsp. polymorphum ATCC 10953]
Length=387

 Score = 52.4 bits (124),  Expect = 3e-05, Method: Compositional matrix adjust.
 Identities = 47/201 (23%), Positives = 87/201 (43%), Gaps = 22/201 (10%)

Query  34   ENKNTDQHLATSRWRKFSREWIRTAKSDSLDISWLKDKDSIDADSLPEPDVLAAEAMGEL  93
            +NKN D        +         A S ++ I    +K S +   +  P++   + + +
Sbjct  107  KNKNVDLLYLYFYLKTIKNNLESIASSTTVKII---NKSSFEKIEISLPNLEIQKKISKK  163

Query  94   VQAL-SELDALMRELGASDEADLQRQLLEEAFGGVKEMSAGKLPEGWVIAPVSTVTTLIR  152
            ++ L + +D    +L    E  L + L    FG +K        + W I  +     +I
Sbjct  164  LELLENNIDFRKNQLNYLKE--LNKSLFTRMFGDIKTND-----KNWKIVKLEKYINIIG  216

Query  153  GVTYKKEQAINYLKDDYLPLIRANNIQNGKFDTTDLVFVPKNLVKESQKISPEDIVIAMS  212
            G   +K       K   +PLIR  NI +G+F +T+LVF+ +N   E  K+ P DI+I+++
Sbjct  217  GYAFKNID----FKSSGIPLIRIGNINSGQFKSTNLVFIEENKKFEKFKVFPNDILISLT  272

Query  213  S-------GSKSVVGKSAHQH  226
                    G+  ++G S  ++
Sbjct  273  GTVGKDDYGNACILGDSYSEY  293
```

c.

```
ref|ZP_00682365.1| similar to Restriction endonuclease S subunits [Xylella fastidiosa
Ann-1]
Score =  122 bits (306),  Expect = 2e-26, Method: Compositional matrix adjust.
 Identities = 78/158 (49%), Positives = 91/158 (57%), Gaps = 28/158 (17%)

Query  64   NDYNLSVSSYVEAKDNREIIDIAELNAELKTTVSKIDQLRKDIDAIVAEIEGCEVQKMSE  123
             D  L      +A  N E  D AE N    T ++ ID+L   I A+  E
Sbjct  95   RDALLRFGGSTDASGNGE--DGAERNQWKPTGINWIDEL---IAALCPE-----------  138

Query  124  MSYLEKLLDGVEVEWLPLGEITKYEQPTKYLVKAKDYHDTYTIPVLTAGKTFILGYTNET  183
                     GVE + L  GE+  YEQP KYLV +  Y ++Y  PVLTAG+TFILGYT+ET
Sbjct  139  ---------GVEFKML--GELLDYEQPGKYLVASTAYDNSYWTPVLTAGQTFILGYTDET  187

Query  184  HGIYQAS-KAPVIIFDDFTTANKWVDFDFKAKSSAMKM  220
             GIY AS + PVIIFDDFTTA KWVDF FKAKSSAMKM
Sbjct  188  SGIYAASPQEPVIIFDDFTTAFKWVDFPFKAKSSAMKM  225
Red is the M.EcoR124I sequence
```

Supplementary figure 5 c cont.

```
ref|YP_001181695.1| restriction modification system DNA specificity domain [Shewanella
putrefaciens CN-32]
Score =  118 bits (296),  Expect = 2e-25, Method: Compositional matrix adjust.
 Identities = 74/149 (49%), Positives = 96/149 (64%), Gaps = 14/149 (9%)

Query  83   IDIAELNAELKTTVSKI-DQLRKDIDAIVAEIEGCEVQKMSEMSYL----EKLLD----G  133
            IDI  L  + K  ++KI     K I      I+  + QK + M  L    ++LLD
Sbjct  157  IDIPPLPEQRK--IAKILSTWDKAISTTERLIDNSKQQKKALMQQLLTAKKRLLDDSGKP  214

Query  134  VEVEW--LPLGEITKYEQPTKYLVKAKDYHDTYTIPVLTAGKTFILGYTNETHGIYQASK  191
             E EW  + LG++  Y+QPT YLVK+ DY + Y+IPVLTAGKTFILGY+NE  GI++  +
Sbjct  215  FEGEWTKVELGKLLDYKQPTPYLVKSTDYSNEYSIPVLTAGKTFILGYSNENFGIFE-EE  273

Query  192  APVIIFDDFTTANKWVDFDFKAKSSAMKM  220
             P IIFDDFTTA+K+VDF FKAKSSAMK+
Sbjct  274  LPAIIFDDFTTASKFVDFPFKAKSSAMKI  302
Red is the M.EcoR124I sequence
```

```
ref|NP_858129.1| putative type i restriction hsds subunit [Lactobacillus delbrueckii
subsp. lactis]
Score =  111 bits (278),  Expect = 3e-23, Method: Compositional matrix adjust.
 Identities = 62/141 (43%), Positives = 90/141 (63%), Gaps = 9/141 (6%)

Query  85   IAELNAELKTTVSKIDQLRKDIDAIVAEIEGCEVQKM--SEMSYLEKLLDGVEVEW--LP  140
            IA   +EL T ++  ++ ++ +  + + +    +QKM   +  Y     +G   EW
Sbjct  171  IANFFSELDTAITLHEEKKQQLKCLKSAL----LQKMFAYKSGYPAIRFEGFSDEWEQCK  226

Query  141  LGEITKYEQPTKYLVKAKDYHDTYTIPVLTAGKTFILGYTNETHGIYQAS-KAPVIIFDD  199
            LGE+  YEQPTKY+VK+ +Y D +  PVLTAGK+F+LGYT+E  GI  A+ + PV+IFDD
Sbjct  227  LGEVFNYEQPTKYIVKSTEYDDNFNTPVLTAGKSFLLGYTDEISGIKNATVENPVVIFDD  286

Query  200  FTTANKWVDFDFKAKSSAMKM  220
            FTT + +VDF FK KSSAMK+
Sbjct  287  FTTDSHYVDFPFKIKSSAMKL  307
Red is the M.EcoR124I sequence
```
